# Predicted Benign and Synonymous Variants in *CYP11A1* Cause Primary Adrenal Insufficiency Through Missplicing

**DOI:** 10.1210/js.2018-00130

**Published:** 2018-10-30

**Authors:** Avinaash Maharaj, Federica Buonocore, Eirini Meimaridou, Gerard Ruiz-Babot, Leonardo Guasti, Hwei-Ming Peng, Cameron P Capper, Neikelyn Burgos-Tirado, Rathi Prasad, Claire R Hughes, Ashwini Maudhoo, Elizabeth Crowne, Timothy D Cheetham, Caroline E Brain, Jenifer P Suntharalingham, Niccolò Striglioni, Bilgin Yuksel, Fatih Gurbuz, Sangay Gupta, Robert Lindsay, Robert Couch, Helen A Spoudeas, Tulay Guran, Stephanie Johnson, Dallas J Fowler, Louise S Conwell, Aideen M McInerney-Leo, Delphine Drui, Bertrand Cariou, Juan P Lopez-Siguero, Mark Harris, Emma L Duncan, Peter C Hindmarsh, Richard J Auchus, Malcolm D Donaldson, John C Achermann, Louise A Metherell

**Affiliations:** 1 Centre for Endocrinology, William Harvey Research Institute, Queen Mary University of London, London, United Kingdom; 2 Genetics and Genomic Medicine, UCL Great Ormond Street Institute of Child Health, University College London, London, United Kingdom; 3 Division of Metabolism, Endocrinology, and Diabetes, Department of Internal Medicine, University of Michigan, Ann Arbor, Michigan; 4 Department of Pharmacology, University of Michigan, Ann Arbor, Michigan; 5 Department of Paediatric Endocrinology and Diabetes, Bristol Royal Hospital for Children, University Hospitals Bristol NHS Foundation Trust, Bristol, United Kingdom; 6 Institute of Genetic Medicine, Newcastle University, Newcastle, United Kingdom; 7 Department of Pediatric Endocrinology and Diabetes, Cukurova University, Adana, Turkey; 8 Department of Pediatrics, Hull Royal Infirmary, Hull, United Kingdom; 9 Institute of Cardiovascular and Medical Sciences, British Heart Foundation Glasgow Cardiovascular Research Centre, University of Glasgow, Glasgow, United Kingdom; 10 Division of Pediatric Endocrinology, Department of Pediatrics, University of Alberta, Edmonton, Alberta, Canada; 11 Department Pediatric Endocrinology and Diabetes, Marmara University, Istanbul, Turkey; 12 Lady Cilento Children’s Hospital, Brisbane, Queensland, Australia; 13 University of Queensland, Brisbane, Queensland, Australia; 14 Institute of Health and Biomedical Innovation, Faculty of Health, Queensland University of Technology, Brisbane, Queensland, Australia; 15 Department of Endocrinology, l’Institut du Thorax, Centre Hospitalier Universitaire de Nantes, Nantes, France; 16 INSERM UMR 1087, CNRS UMR 6291, l'Institut du Thorax, Université de Nantes, Nantes, France; 17 Pediatric Endocrinology Unit, Children’s Hospital, Institute of Biomedical Research in Malaga, Málaga, Spain; 18 Department of Endocrinology and Diabetes, Royal Brisbane and Women's Hospital, Brisbane, Queensland, Australia; 19 Faculty of Medicine, University of Queensland, Brisbane, Queensland, Australia; 20 Department of Paediatrics, University College London Hospitals, London, United Kingdom; 21 Section of Child Health, Glasgow University School of Medicine, Glasgow, United Kingdom

**Keywords:** Addison disease, silent variant, side chain cleavage enzyme, cytochrome p450scc, CYP11A1

## Abstract

Primary adrenal insufficiency (PAI) is a potentially life-threatening condition that can present with nonspecific features and can be difficult to diagnose. We undertook next generation sequencing in a cohort of children and young adults with PAI of unknown etiology from around the world and identified a heterozygous missense variant (rs6161, c.940G>A, p.Glu314Lys) in *CYP11A1* in 19 individuals from 13 different families (allele frequency within undiagnosed PAI in our cohort, 0.102 vs 0.0026 in the Genome Aggregation Database; *P* < 0.0001). Seventeen individuals harbored a second heterozygous rare disruptive variant in *CYP11A1* and two had very rare synonymous changes in *trans* (c.990G>A, Thr330 = ; c.1173C>T, Ser391 =). Although p.Glu314Lys is predicted to be benign and showed no loss-of-function in an *Escherichia coli* assay system, *in silico* and *in vitro* studies revealed that the rs6161/c.940G>A variant, plus the c.990G>A and c.1173C>T changes, affected splicing and that p.Glu314Lys produces a nonfunctional protein in mammalian cells. Taken together, these findings show that compound heterozygosity involving a relatively common and predicted “benign” variant in *CYP11A1* is a major contributor to PAI of unknown etiology, especially in European populations. These observations have implications for personalized management and demonstrate how variants that might be overlooked in standard analyses can be pathogenic when combined with other very rare disruptive changes.

The first step in steroidogenesis involves cleavage of cholesterol to pregnenolone by the cytochrome P450 side chain cleavage enzyme (CYP11A1). CYP11A1 is encoded by *CYP11A1* and is expressed in key steroidogenic tissues such as the adrenal gland and gonads. Further tissue-specific enzymatic steps lead to production of all other steroid hormones. In the adrenal gland, this ultimately results in the production of glucocorticoids (cortisol) and mineralocorticoids (aldosterone) and weak androgens, and in the gonads, the production of sex steroids (estrogen and testosterone) [1].

Complete loss of CYP11A1 prevents biosynthesis of all steroid hormones and was predicted to be incompatible with life owing to the inability of the placenta to maintain a pregnancy without progesterone production from fetally derived tissue [2]. However, it has become clear that biallelic mutations in *CYP11A1* are compatible with survival to term. Defects in *CYP11A1* can cause a range of phenotypes: from classic CYP11A1 deficiency with severe disruption of adrenal and gonadal steroidogenesis, causing salt-losing adrenal insufficiency and gonadal hormone deficiency, to very mild phenotypes in which only glucocorticoids are affected [OMIM (Online Mendelian Inheritance in Man) no. 613743] [3–14] (Supplemental Table 1).

Massively parallel sequencing technologies have expedited discovery of disease-causing variants. However, assigning causality to the identified variants can be complex. When filtering for causal variants, synonymous changes (which do not affect amino acid coding) could be discarded, without considering their allele frequency. In addition, variants predicted to be “benign” at the protein level could also be deselected. Furthermore, splice site changes can only be considered if they alter the canonical GT…AG motifs bordering introns. Such stringency could result in missing pathogenic and clinically relevant variants.

In the present study, we have investigated a large cohort of children and young adults with primary adrenal insufficiency (PAI) of unknown etiology. We found that compound heterozygous variants in *CYP11A1* involving rs6161 (c.940G>A; p.Glu314Lys) were surprisingly common and that altered splicing should be considered when predicted benign or very rare synonymous changes are found.

## 1. Material and Methods

### A. Subjects and Sequencing

The main focus of the present study was to assess *CYP11A1* in subjects with PAI of unknown etiology. The inclusion criteria included evidence of low cortisol, an attenuated cortisol response on cosyntropin stimulation testing, and elevated ACTH, with clinical signs of cortisol insufficiency and hyperpigmentation [15, 16] (Table 1). Some subjects also had elevated plasma renin activity and low aldosterone and/or electrolyte disturbances (hyponatremia, hyperkalemia) consistent with mineralocorticoid insufficiency.

**Table 1.** Clinical Presentation of 19 Individuals With *CYP11A1* Mutations

Subject No.	Sex	Age at Diagnosis*^a^*	Presentation	Replacement	ACTH at Presentation, pg/mL	Cortisol at Presentation (Peak Stimulated, nmol/L	Na/K, mEq/L	Plasma Renin Activity, ng/mL/h (Unless Stated Otherwise)	Aldosterone (pmol/L)	Puberty	FSH (IU/L), LH (IU/L), Testosterone (nmol/L), Estradiol (pmol/l)	Comment
1	M	10 y	Hypoglycemic convulsions, vomiting, hyperpigmentation	GC	149	234 (peak 118)	142/4.4	2.0 (<3.0)	300	Normal	FSH 3.4, LH 4.1, testosterone 14.8 (21 y)	Original diagnosis, ketotic hypoglycemia; reported by Chan *et al.* [15]
2	M	9 mo	Hypoglycemia (ketotic)	GC	1002	93	N	N	ND	Delayed then progressed	N	Fertile
3	F	11 mo	Failure to thrive, anorexia, hyperpigmentation	GC	>1500	190	N	ND	496	Normal	ND	Brother died at age 3 y with similar features
4	M	11 mo	Pneumonia, hypoglycemia, collapse	GC, MC	155 (had been receiving treatment)	90 (peak 264)	132/4.0	5.3	ND	Normal	FSH 9.0, LH 7.4, testosterone 20.3 (16 y)	None
5A	F	16 y	Secondary amenorrhea or galactorrhea, pituitary corticotrope adenoma, hyperpigmentation	GC	3354	108 (peak 154)	ND	2.4	1136	Normal (secondary amenorrhea)	N	Pituitary macroadenoma, prolactinemia; reported by Benoit *et al.* [16]
5B	F	14 y*^b^*	Investigated because of diagnosis in sister	GC (24 y)	400	276 (peak 303)	ND	N	416	Normal	ND	None
6	M	4 y	Recurrent illnesses, hyperpigmentation	GC, MC	1147	252 (peak 79)	133/5.4	ND	ND	Delayed then progressed	FSH 18.3, LH 9.1 (16 y); FSH 9.0, LH 6.1, testosterone 13.2 (25 y)	None
7	M	7 y	Recurrent illnesses, hyperpigmentation	GC, MC	1091	31	N	89.4/202 mU/L	<100	NA (prepubertal)	NA	None
8A	M	9 y	Convulsions, hyperpigmentation, diagnosis in younger brother	GC, MC	6128 (23 y)	339 (peak 389)	137/3.5	9.7 (23 y); 83.5 mIU/L (<40) (38 y)	ND	Delayed then progressed	FSH 13.8, LH 5.8, testosterone 24.7 (38 y)	None
8B	M	8 y	Convulsions, hyperpigmentation, diagnosis in younger brother	GC, MC	264 (17 y; with treatment)	278 (peak 301)	141/3.6	10.2 (21 y)	ND	Delayed then progressed	FSH 9.3, LH 5.4, testosterone 33.3 (36 y)	None
8C	M	1.5 y	Febrile convulsions, hypoglycemia, hyperpigmentation	GC, MC	4356	174 (peak 178)	129/5.9	20.9 (<2.0) (17 y); 119.2 mIU/L (<40) (32 y)	ND	Normal	FSH 41.2, LH 33.9, testosterone 27.2 (32 y)	None
9	M	2 y	Hypoglycemia, salt-wasting, hyperpigmentation	GC, MC	463	157	127/6.8	ND	<100	Normal	FSH 14, LH 15, total testosterone 7 ng/mL (15 y)	None
10A	F	15 y	Investigated because diagnosis in brother	GC	67	129 (peak 279)	141/4.2	2.5 (0.5–3.1)	380	Normal	N	None
10B	M	5 y	Recurrent illnesses, ketotic hypoglycemia, hyperpigmentation	GC	385	116 (172)	N (Na128 vomiting)	3.1 (<7.0)	140	NA (prepubertal	NA	None
11A	F	4 y	Hypoglycemic seizure, cardiac arrest, died	GC	>1250	73	ND	ND	ND	NA (died)	NA (died; normal ovaries on autopsy)	Autopsy: small adrenal glands with normal zona glomerulosa and atrophied zona fasciculata
11B	F	2 y	Hyperpigmentation, diagnosis in elder sister	GC	686	Peak 55	140/4.0	45 mU/L (3–35)	155	Normal	FSH 3.6, LH 3.2, estradiol 200 (14 y)	Hypertension treated with captopril; small adrenal glands on CT
11C	M	11 mo	Investigated because diagnosis in sister	GC	350	226 (276)	137/4.1	70 mU/L (3–35) (14 y)	227 (14y)	Normal	FSH 14, LH 4.5, testosterone 14 (14 y)	Hypertension treated with Ramipril; also treated for steroid-responsive nephrotic syndrome (minimal change disease)
12	F	6 mo	Hyperpigmentation	GC	2896	14 (14)	ND	54 ng/L (<150)	97	NA (prepubertal)	NA	None
13	F	3 y	Hypoglycemia, hyperpigmentation, lethargy	GC, MC	1487	64 (with blood glucose 1.6 mmol/L)	131/4.3	27.7	137	NA (prepubertal)	NA	None

ACTH, pg/mL × 0.22 for pmol/L (normal range, 10–60 pg/mL); cortisol, nmol/L × 0.036 for μg/dL (normal peak stimulated value >550 nmol/L); plasma renin activity, pmol/mL/h × 1.3 for ng/mL/h; aldosterone, pmol/L × 0.36 for pg/mL; testosterone, nmol/L × 28.9 for ng/dL; estradiol, pmol/L × 0.27 for pg/mL. Additional normal ranges are presented, as well as age at sampling. Some variations could have occurred because of different assay methods and age of the patient.

Abbreviations: F, female; GC, glucocorticoid; M, male; MC, mineralocorticoid; N, within normal range; No., number; NA, not applicable; ND, not done.

^a^ Age at diagnosis shown corresponded to age at which investigations were undertaken unless indicated.

^b^ Regular replacement therapy was initiated at a later age.

The exclusion criteria were an established biochemical and/or genetic diagnosis, such as other forms of congenital adrenal hyperplasia (*e.g.,* 21-hydroxylase deficiency, 11*β*-hydroxylase deficiency, 3*β*-hydroxysteroid-dehydrogenase deficiency type 2), autoimmune adrenalitis, metabolic disorders or physical cause of adrenal insufficiency (*e.g.* hemorrhage, infection), or known genetic causes of familial glucocorticoid deficiency or adrenal hypoplasia. Individuals with isolated hypospadias, 46,XY disorders of sex development, or intrauterine growth restriction (<2 SD) with associated adrenal insufficiency were also excluded.

The patients were recruited from three main cohorts: (i) The “Barts/Royal London Hospital/Queen Mary University of London,” which included 43 individuals with PAI of unknown etiology, who were assessed by exome sequencing, targeted panel testing, or direct Sanger sequencing; (ii) the “UCL/Great Ormond Street Institute of Child Health” cohort, which included 25 individuals with PAI of unknown etiology, who were assessed by targeted panel testing; and (iii) the “Turkish” cohort, which included 9 individuals with PAI of unknown etiology, who were assessed by targeted panel and exome sequencing. Using this approach, the prevalence of *CYP11A1* c.940G>A as a cause of PAI in a cohort (n = 77) with no current diagnosis could be determined (Supplemental Table 2).

To establish the prevalence of CYP11A1 c.940G>A as a cause of PAI in these cohorts in general, the total numbers of individuals recruited over the years were calculated (n = 395). Although considerable overlap was present in the clinical features, the “Barts/Royal London Hospital/Queen Mary University of London” cohort (total n = 256) focused more on classic “familial glucocorticoid deficiency” [*e.g.,MC2R*, *MRAP*, *NNT*, *AAAS*, *STAR* (steroidogenic acute regulatory protein), *MCM4*]. The “UCL/Great Ormond Street Institute of Child Health” cohort (total n = 57) focused more on adrenal hypoplasia (*e.g.,NR0B1*, with potential nonclassic *STAR*, *NNT*, and severe *MC2R* among the diagnoses; almost 80% of the children were receiving mineralocorticoid replacement). The “Turkish” cohort (total n = 82) included a range of diagnoses recently reported (*e.g.,MC2R*, *CYP11A1*, *MRAP*, *NNT*) and represented individuals and families with high consanguinity [3]. For the present analysis, individuals with hypospadias or 46,XY disorders of sexual development were excluded, and children with the classic forms of congenital adrenal hyperplasia (*e.g.,* 21-hydroxylase), autoimmune disorders, or physical causes would not have been referred. Finally, one family (three individuals) from Australia was included because the diagnosis had been reached by exome sequencing. However, that kindred was not included in prevalence data because they did not form a part of a cohort.

#### A-1. DNA samples

Studies were performed with the approval of the local ethics committees (Outer North East London research ethics committee, reference number, 09/H0701/12; London-Chelsea National Research Ethics Service committee, reference number, 13/LO/0224; London-Bloomsbury National Research Ethics Service committee, reference number, 07/Q0508/24; the Mater Hospital ethics committee, reference number, 1931C). After ethical approval and informed consent from the individuals or their families, genomic DNA was extracted from whole blood of the affected individuals, and their parents and unaffected siblings, if available.

#### A-2. Sequencing

Whole exome sequencing was used for subjects 1, 11A, 11B, and 11C; *CYP11A1* alone was sequenced for subjects 12 and 13. All other subjects were analyzed using the HaloPlex targeted capture array (Agilent Technologies, Inc., Santa Clara, CA) [3, 15] (see section A-4). Sanger sequencing was used to confirm segregation of the variants in the kindreds in which the parents and/or unaffected siblings were available.

#### A-3. Exome sequencing

Subject 1 was sequenced as described previously [15]. For individuals 11A, 11B, and 11C, exome sequencing libraries were constructed using the Nextera Rapid Capture Exome (Illumina, Inc., San Diego, CA), according to the manufacturer’s recommendations. In brief, 50 ng of genomic DNA was tagmented (fragmented and adapter sequences added) using the Nextera transposomes. Tagmented samples were purified, and the fragment size was confirmed using the 2100 Bioanalyser (Agilent Technologies, Inc.). The libraries were denatured into single-strand DNA, and biotin-labeled probes specific to the target regions were used for Rapid Capture hybridization. The pool was enriched for the desired regions by adding streptavidin beads that bind to the biotinylated probes. Biotinylated DNA fragments bound to the streptavidin beads were magnetically pulled down from the solution. The enriched DNA fragments were then eluted from the beads and hybridized for a second Rapid Capture hybridization. A second magnetic bead cleanup was performed. The final libraries were analyzed using the 2100 Bioanalyzer and DNA 1000 chip kit (Agilent Technologies, Inc.) to determine the quantity and size of the enriched fragments.

Massive parallel sequencing was performed with six samples per flow cell lane via the Illumina HiSeq2000 platform and version 3 SBS reagents to generate 100 bp paired-end reads. After demultiplexing, the Illumina Data Analysis Pipeline software (CASAVA, version 1.8.2) was used for initial base calling. Sequence data were aligned to the current build of the human genome (UCSC Genome Browser, hg19, released February 2009) via the Novoalign alignment tool (version 2.08.02 1). Sequence alignment files were converted using the SAMtools, version 0.1.14, and Picard tools, version 1.42. Single nucleotide polymorphisms (SNPs) and indels were called with the Genome Analysis Toolkit, version 5506, and annotated using ANNOVAR.

Further analysis of sequence data was performed with custom scripts using R and Bioconductor. We retained good-quality SNPs and indels (minimum depth of coverage for SNP calling >10-fold for homozygous SNPs; >15-fold for heterozygous SNPs). Additionally, we used variants that had passed the Genome Analysis Toolkit Variant quality score recalibration.

The remaining SNPs and indels were assessed according to the prediction of potentially damaging consequence [“nonsynonymous single nucleotide variant” (SNV) “splicing,” “frameshift substitution,” “stopgain SNV,” “stoploss SNV”] using both RefSeq and UCSC transcripts. Further filtering excluded SNPs with a minor allele frequency (MAF) >0.01 observed in the National Center for Biotechnology Information dbSNP, release 137 (available at: www.ncbi.nlm.nih.gov/SNP/), 1000 Genomes, 1000 Genomes small indels (called the DINDEL program), the SNPs of 46Genomes release by Complete Genomics, and other whole exomes from >3000 control samples run internally using similar capture technology. Variants not present in any of these databases were considered novel.

#### A-4. HaloPlex targeted gene panel and next generation sequencing

A custom HaloPlex DNA target enrichment panel (Agilent Technologies, Inc., Santa Clara, CA) was designed (SureDesign) to capture 160 known and candidate genes involved in adrenal development and function. All coding exons and 100 base pairs of the intronic flanking sequence were included. The panel covered known genes potentially causing PAI, congenital adrenal hyperplasia-related genes, potential syndrome-related genes, and candidate genes based on data from biochemical and/or biological pathways, mouse models of adrenal dysfunction, and gene expression [3].

Sequence capture was performed according to the HaloPlex Target Enrichment Protocol, version D.5 (Agilent Technologies, Inc., Santa Clara, CA) for Illumina sequencing. Patient genomic DNA aliquots (225 ng) were processed in batches of 24 samples with an enrichment control DNA sample as a positive control. Sequencing was performed using a MiSeq next generation sequencer (Illumina, Inc., San Diego, CA). Sequence alignment and variant calling were performed using SureCall, version 2.0, software (Agilent Technologies, Inc., Santa Clara, CA).

#### A-5. Sanger sequencing

Potential disease causing variants were confirmed using PCR and Sanger sequencing. *CYP11A1* exons of interest, including intronic boundaries, were amplified by PCR using specific primers (Supplemental Table 3). The reaction mixture contained 100 ng DNA template, 1 × PCR buffer, 200 µM for each deoxyribonucleotide triphosphate (dNTP), 200 nM for each primer, and 1 U Taq DNA polymerase (Sigma-Aldrich, St. Louis, MO). The cycling conditions were 95°C for 5 minutes (1 cycle); 95°C for 30 seconds, 55°C for 30 seconds, and 72°C for 30 seconds (30 cycles); and 72°C for 5 minutes. The PCR products were visualized on 1% agarose gel and sequenced using the ABI Prism Big Dye sequencing kit and an ABI 3700 automated DNA sequencer (Applied Biosystems, Foster City, CA), in accordance with the manufacturer’s instructions.

#### A-6. Sequence interpretation

Variants were considered highly likely to be pathogenic if they segregated with the phenotype with an appropriate inheritance pattern within families, were determined damaging or likely damaging by several bioinformatic prediction models (Ensembl Variant Effector Predictor; SIFT; PolyPhen-2; and Mutation Taster; see the Appendix) and/or if they had been reported in association with adrenal insufficiency previously. In addition, other missense and synonymous changes with a MAF <3% in the Exome Aggregation Consortium (ExAC) browser (Exome Aggregation Consortium, Cambridge, MA) were considered.

#### A-7. *In Silico* Analysis of Variants

MAFs were determined from the ExAC and Genome Aggregation Database (gnomAD) browsers. The functionality of the variants was assessed at all levels using the variant effect predictor and MutationTaster. Splice function was assessed using Human Splicing Finder, version 3.0, SpliceAid, and ESEfinder (see the Appendix).

### B. Statistical Analysis

The difference in allele frequencies between subjects (counting one proband per family to avoid cascade testing bias) and controls (MAF from gnoMAD non-Finnish Europeans, determined from ExAC data) was measured using *χ*^2^ test with Yates’ correction.

### C. *In Vitro* Splicing Assay

An *in vitro* splicing assay was designed using the commercially obtained Exontrap cloning vector pET01 (MoBiTec GmbH, Göttingen, Germany) containing an intronic sequence interrupted by a multiple cloning site. DNA fragments of interest were amplified using a standard PCR protocol and specifically designed primers (Supplemental Table 4) containing a restriction enzyme target site for *Xba*I. The cycling conditions were as follows: 95°C for 5 minutes, 15 × [95°C for 30 seconds, 70°C for 30 seconds (−1°C per cycle), 72°C for 30 seconds], 30 × (95°C for 30 seconds, 55°C for 30 seconds, 72°C for 30 seconds), and 72°C for 5 minutes. PCR products were sequenced as above (section A-5), column purified using the QIAquick^®^ PCR Purification Kit according to the manufacturer’s protocol, and cloned into the Exontrap cloning vector pET01 (MoBiTec GmbH). The cloned sequences were verified by Sanger sequencing to ensure the fragment was in the correct orientation using pET01 specific primers [ET PRIM 06 (forward) GCGAAGTGGAGGATCCACAAG and ET PRIM 07 (reverse) ACCCGGATCCAGTTGTGCCA]. Wild-type (pET01-WT) or pET01 mutant plasmids were transfected into HEK293 cells using Lipofectamine^®^ reagent (Thermo Fisher Scientific, Waltham, MA). Total RNA, obtained from cells 24 hours after transfection, was subjected to RT-PCR to generate cDNA with primer GATCCACGATGC (MoBiTec GmbH) and amplifying with primers within the 5ʹ and 3ʹ exons in the pET01 vector primer 02 (GAGGGATCCGCTTCCTGGCCC) and primer 03 (reverse sequence CTCCCGGGCCACCTCCAGTGCC; Supplemental Table 5). Amplification products were assessed on a 2% agarose gel.

### D. Fibroblast Isolation, Culture, and Expansion

Fibroblast isolation and culture was performed as described previously [17]. In brief, a 4-mm punch biopsy from the upper arm (patient 1 and his parents) was immediately incubated with isolation medium [DMEM supplemented with 10% (v/v) fetal bovine serum (FBS), and 1% penicillin/streptomycin (P/S)] in a 15-mL falcon tube. Next, 1-mm^2^ cubes of skin sample were digested using DMEM/high glucose, 20% FBS (v/v), 0.25% collagenase type I, 0.05% DNAse-I, and 1% P/S (Sigma-Aldrich) at 37°C overnight in a 15-ml falcon tube. After centrifugation for 5 minutes at 1000 rpm, the pellet was resuspended in 5 mL of isolation medium before plating in gelatin coated T25 flasks. The cells were kept in human fibroblast media [DMEM/high glucose with sodium pyruvate and l-glutamine, 20% FBS (v/v) and 1% P/S].

### E. RNA Extraction and PCR Analysis

Total RNA was purified using an RNeasy Mini Kit (model no. 74106; Qiagen, Valencia, CA). RNase-Free DNase Set (model no. 79254; Qiagen) was used to eliminate genomic DNA contamination. Next, 50 ng of RNA was incubated with 60 ng/µL of random primers for 5 minutes at 70°C, followed by incubation with 500 µM dNTPs, 40 U of RNAse inhibitor, 200 U of Moloney murine leukemia virus RT and 1× Moloney murine leukemia virus buffer for 10 minutes at 25°C, 90 minutes at 42°C, and 15 minutes at 70°C to generate cDNA. PCR amplifications were performed using 2 ng of cDNA, dNTPs of 200 µM, and Q5 DNA polymerase (model no. M0491; New England Biolabs, Inc.) using primers in exons 3 and 6 of *CYP11A1* (Supplemental Table 6).

### F. Protein Expression in *Escherichia coli* and Purification

The F2 construct of human *CYP11A1*, adrenodoxin (Adx; ferredoxin), and ferredoxin reductase was provided by Professor Walter L. Miller (University of California, San Francisco, CA) [18]. From the construct, three different cDNAs were constructed separately using PCR. A cDNA encoding human *CYP11A1* in pcDNA3 was amplified by PCR to remove the 39-amino acid *N*-terminal mitochondria-targeting sequence and to add a His6-tag at the C-terminus. The primers were 11A1-sense 5′-ATACATATGGCGTCTACCCGTTCTCCT-CGCCCCTTCAATGAGAT-3′; 11A1-antisense 5′-AGAATTCTCAGTGATGGTGATGGTG-ATGCTGCTGGGTTGCTTCCT-3′. The PCR products were then cloned into the pET-17b vector via *Nde*I/*Eco*RI restriction sites. The cDNA for ferredoxin reductase was amplified by PCR to include the His6-tag at the C-terminus and cloned into pET-17b via *Nde*I/*Eco*RI restriction sites. The primers were FdR-sense 5′-ATACATATGGCGTCTACCCAGGAAAAGACCCCACAG-3′; and FdR-antisense 5′-AGAATTCTCAATGGTGATGGTGATGGTGGTGGCCCAGGAGGC-GCA-3′. The sequence coding for mature human Adx (amino acids 62 to 184) was amplified by PCR to include the His6-tag at the *N*-terminus and cloned into pLW01 via *Nco*I/*Eco*RI restriction sites. The primers were Adx-sense 5′-TATACCATGGCACACCATCACCATCACCATTCATCAG-AAGATAAAATAACAGTC-3′, and Adx-antisense 5′-ATGAATTCTCAGGAGGTCTTGCCC-AC-3′. The QuikChange Lightning kit site-directed mutagenesis (Agilent Technologies, Inc.) was used to generate the p.Glu314Lys variants for CYP11A1 in both pcDNA3 and pET-17b. The primers were sense 5′-GCAAGATGTCCTTCAAGGACATCAAGGCC-3′; and antisense 5′-GGCCTTGATGTCCTTGAAGGACATCTTGC-3′. Sanger sequencing of plasmid constructs confirmed the intended site-specific mutations and ensured the lack of other substitutions. WT and Glu314Lys plasmid DNAs were isolated using a Qiagen Maxi Prep kit.

Human Adx was expressed in *E. coli* strain C41(DE3) and purified as described previously [19]. Human ferredoxin reductase [20], CYP11A1 WT [19], and Glu314Lys were expressed in C41(DE3) with pGro7 (Thermo Fisher Scientific) and purified as described previously [21]. In brief, Fernbach flasks containing 1 L of Terrific Broth (supplemented with 0.5 mM 5-aminolevulinic acid for CYP11A1; Sigma-Aldrich) with 100 µg/mL ampicillin (and 20 µg/mL chloramphenicol for pGro7; Sigma-Aldrich) were inoculated with 20 mL of an overnight preculture. The cells were grown at 37°C with shaking at 250 rpm until the A_600_ reached 1.0 to 1.4 AU, at which time the culture was induced with 0.4 mM isopropyl *β*-D-1-thiogalactopyranoside (supplemented with 4 g/L arabinose for ferredoxin reductase and P450s) and grown for 20 to 48 hours at 28°C. After cell lysis with French press in buffer A (sterile phosphate-buffered saline containing 20% glycerol for ferredoxin reductase and CYP11A1), the recombinant P450 proteins were solubilized using 1% cholate (Chem-Impex International) and 0.5% NP-40. After centrifugation at 70,000*g* for 18 minutes, the supernatant was mixed with 3 to 5 mL Ni-NTA affinity resin, and polyhistidine-tagged proteins were eluted with 10 mL of 250 mM imidazole, followed by buffer exchange using PD-10 columns. Purified CYP11A1 preparations showed a specific content of 8 to 12 nmol P450/mg protein with 3% to 10% P420.

### G. Reconstituted Enzyme Assays

In a 2-mL polypropylene tube, purified human CYP11A1 (10 pmol, WT or Glu314Lys) was mixed with an equal amount of ferredoxin reductase, 40-fold molar excess of Adx, and dilauroyl phosphatidyl choline in <10 µL volume and incubated for 5 minutes. The reaction mixture was then diluted to 0.2 mL with 50 mM HEPES buffer (pH 7.4), 4 mM MgCl_2_, 0.2% Tween 20, and substrates 22*R*-hydroxycholesterol (in ethanol) or cholesterol (in methyl-*β*-cyclodextrin inclusion complexes). The resulting mixture was preincubated at 37°C for 3 minutes before adding NADPH (1 mM) and incubating at 37°C for another 20 minutes. The reaction mixture was extracted with 1 mL dichloromethane, and the organic phase was dried under nitrogen flow. The steroids were reconstituted in 70 μL of methanol, and a solution of 0.1 mL 100 mM potassium phosphate buffer (pH 7.4) containing 10 μL of cholesterol oxidase (100 U/mL) was added with 3 μL internal standard (1 mM dehydroepiandrosterone). The mixture was incubated at 30°C for 6 hours to convert the product pregnenolone to progesterone and the internal standard to androstenedione. The mixture was extracted with 1 mL of dichloromethane, and the organic layer was dried under nitrogen flow.

#### G-1. Chromatography, data acquisition, and determination of kinetic constants

Reaction products were analyzed using an Agilent 1260 Infinity HPLC system with an ultraviolet detector. Extracted steroid products were dissolved in 20 μL of methanol, and 5 μL injections were resolved with a 50 × 2.1-mm, 2.6-μm, C_8_ Kinetex column (Phenomenex, Torrance, CA), equipped with a guard column at a flow rate of 0.4 mL/min. A methanol/water linear gradient was used: 27% methanol from 0 to 0.5 minute, 39% to 16 minutes, 44% to 20 minutes, 60% to 22 minutes, 71% to 30 minutes, 75% to 30.5 minutes, and 27% to 33 minutes. The steroids progesterone and androstenedione were identified by the retention times of external standards chromatographed at the beginning and end of the experiments, and the data were processed using Laura4 software (LabLogic) and graphed with GraphPad Prism, version 6 (GraphPad Software, San Diego, CA).

#### G-2. Immunoblotting

One day before transfection, HEK-293T cells were plated in six-well plates at 50% confluency. The next day, the cells were transiently transfected with 1 µg plasmid DNA/well and 3 µL FuGENE 6 transfection reagent/well (Promega, Madison, WI). The transfected cells were treated with 25 µM cycloheximide for the specified period, as indicated, and whole cell protein extracts were collected 48 hours after transfection using radioimmunoprecipitation assay buffer (Sigma-Aldrich) supplemented with Mini Protease Inhibitor Tablets (Roche). Lysates were briefly sonicated for ~10 seconds, centrifuged to remove cellular debris, and resolved (25 µg total protein) on a 4% to 20% SDS-PAGE gel (Novex). The proteins were transferred to a polyvinylidene difluoride membrane (Millipore, Billerica, MA), blocked with 5% fat-free milk in Tris-buffered saline with 0.1% Tween-20, and detected by rabbit anti-CYP11A1 at 1:5000 dilution [22] (RRID: AB_2747382 [23]; a generous gift from Walter Miller); mouse anti–glyceraldehyde 3-phosphate dehydrogenase at 1:10,000 dilution (RRID: AB_2107426 [24]) served as a loading control. Immunoreactive bands were visualized on film using horseradish peroxidase-conjugated mouse or rabbit secondary antibodies (1:5000; RRID: AB_330924 [25] and RRID: AB_2099233 [26], respectively) combined with SuperSignal West Chemiluminescence Substrate (Pierce, Thermo Fisher).

#### G-3. Activity assay in intact cells

V79 hamster lung cells were plated in 12-well plates at ~70% confluency. The next day, the cells were transiently transfected with 1 μg/well pcDNA3-*CYP11A1* plasmids (WT or mutations) in triplicate using TransIT-LT1 transfection reagent (Mirus) according the manufacturer’s instructions. At 24 hours after transfection, the cells were incubated with 1 mL of serum-free medium containing 22R-hydroxycholesterol (5 μM) and dehydroepiandrosterone (control steroid, 1 μM). Aliquots of the medium were removed at 6 and 24 hours, and 0.1 mL of each sample was mixed with deuterium-labeled internal standards and extracted with 1 mL methyl tert-butyl ether. Pregnenolone product was converted to the oxime with hydroxylamine in aqueous ammonia and quantified using tandem mass spectrometry, as described previously [27].

## 2. Results

### A. Prevalence of the CYP11A1 c.940G>A Variant in PAI

**Figure 1.**
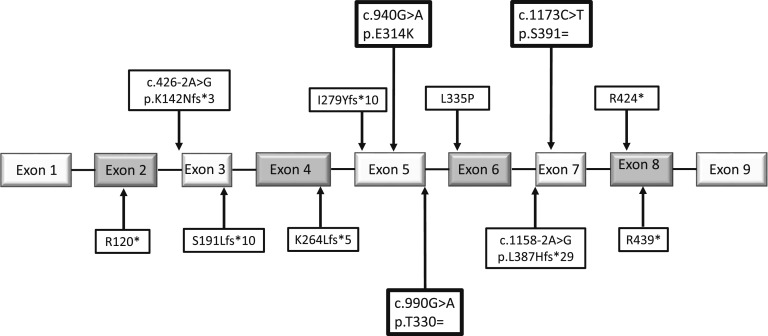
Position of variants in CYP11A1 genomic/pre-mRNA sequence found in this series of patients with PAI. Boxed in bold, the three predicted benign or synonymous variants assessed for their effect on splicing; SNP rs6161 (c.940G>A, p.Glu314Lys) is 110 bp from the start and 51 bp from the end of exon 5, c.990G>A (p.Thr330 =) occurs at the last base of exon 5, and c.1173C>T (p.Ser391 =) is 16 bp from the start of exon 7.

Initially, we undertook whole exome or targeted panel sequencing in 77 individuals or family members with PAI of unknown etiology from the three main cohorts studied (Supplemental Table 2). Sixteen individuals from 12 different families were found to harbor the rs6161 variant in *CYP11A1* (chr15:74635368C>T; c.940G>A), together with another very rare heterozygous variant (Table 2; families 1 to 10, 12, and 13), for an overall prevalence of *CYP11A1* c.940G>A associated with PAI of unknown etiology of 20.8% (16 of 77) and a prevalence of 4.1% (16 of 395) in our cohorts of patients with adrenal insufficiency overall (Supplemental Table 2). Even when only one proband was counted per family to avoid cascade testing bias, the MAF of rs6161 was enriched in our cohort, with a frequency of 0.102 (12 of 59 compared with a MAF of 0.0026 across all gnomAD populations or 0.0042 in non-Finnish Europeans; *χ*^2^*P* < 0.0001). Thus, ~1 in 200 of the population (and 1 in 120 Europeans) are heterozygous carriers of this variant compared with 1 in 5 of the undiagnosed PAI study group. Finally, a further family from Australia with three affected individuals was identified to have this variant, together with a second change, and was included in our series (Table 1; family 11). Consequently, compound heterozygosity of rs6161 with another disruptive variant in *CYP11A1* was identified in 19 individuals from 13 families in total. Validation of the variants and segregation with disease was confirmed by Sanger sequencing in these patients and in their family members, if possible, showing that the variants occurred in *trans* (Table 2; Fig. 1).

**Table 2.** Genetic Diagnoses in 19 Patients With *CYP11A1* Mutations

Subject No.	Genomic Coordinates and Nucleotide Change (Genome Assembly GRCh37.p13)	cDNA Position and Nucleotide Change (Transcript NM_000781)	Protein (Prediction)	dbSNP/HGMD (if Annotated)	Alleles Present in gnomAD; Number Sequenced in gnomAD; MAF gnomAD	Predicted*^a^* and/or Tested Consequence	Segregation	Country	Sequencing Method
All	74635368C>T	940G>A	E314K	rs6161	710; 277190(4 homozygotes);0.002561	Missense*^a^*/skipping of exon 5 = p.A277Dfs*11	Yes	Various	Various
Variants in *trans* with rs6161									
1	74635318C>T	990G>A	T330 =	NA	Not seen;0	Silent*^a^*/skipping of exon 5 p.A277Dfs*11 and NMD	Yes	United Kingdom	WES
2	74635473delT	835delA	I279Yfs*10	rs757299093/CD050132	7; 277156;4.061e-6	Early stop*^a^*	Yes	United Kingdom	HaloPlex
3	74631031G>A	1315C>T	R439*	rs755975808	2; 277174;7.216e-6	Early stop*^a^*	Yes	Spain	HaloPlex
4	74631641G>A	1173C>T (ND)	S391 =	rs751829641	6; 277178;2.165e-5	Silent*^a^*/skipping exon 7 = p.L387Hfs*29	ND	United Kingdom	HaloPlex
5A	74632081A>G	1004T>C	L335P	NA	Not seen;0	Missense*^a^*	Yes	France	HaloPlex
5B	74632081A>G	1004T>C	L335P	NA	Not seen;0	Missense*^a^*	Yes	France	HaloPlex
6	74637586T>C	c.426-2A>G	exon 3 skip	rs754329273	2; 245202;8.157e-6	Skipping of exon 3*^a^*, resulting in p.K142Nfs*3	ND	United Kingdom	HaloPlex
7	74631076G>A	1270C>T	R424*	rs762412759	5; 277032;1.805e-5	Truncation/NMD*^a^*	ND	United Kingdom	HaloPlex
8A	74636157_74636169del	c.790_802del	K264Lfs*5	NA	Not seen;0	Early stop*^a^*	Yes	United Kingdom	HaloPlex
8B	74636157_74636169del	c.790_802del	K264Lfs*5	NA	Not seen;0	Early stop*^a^*	Yes	United Kingdom	HaloPlex
8C	74636157_74636169del	c.790_802del	K264Lfs*5	NA	Not seen;0	Early stop*^a^*	Yes	United Kingdom	HaloPlex
9	74631658T>C	c.1158-2A>G	exon 7 skip	NA	Not seen;0	Skipping of exon 7 resulting in p.L387Hfs*29*^a^*	ND	Turkey	HaloPlex
10A	74640308G>A	c.358C>T	R120*	NA	2; 246224;8.123e-6	Early stop	ND	United Kingdom	HaloPlex
10B	74640308G>A	c.358C>T	R120*	NA	2; 246224;8.123e-6	Early stop	ND	United Kingdom	HaloPlex
11A	74635473delT	c.835delA	I279Yfs*10	rs757299093/CD050132	7; 277156;4.061e-6	Early stop*^a^*	Yes	Australia	WES
11B	74635473delT	c.835delA	I279Yfs*10	rs757299093/CD050132	7; 277156;4.061e-6	Early stop*^a^*	Yes	Australia	WES
11C	74635473delT	c.835delA	I279Yfs*10	rs757299093/CD050132	7; 277156;4.061e-6	Early stop*^a^*	Yes	Australia	WES
12	74637444dupG	c.566dupC	S191Lfs*10	NA	Not seen;0	Early stop	ND	Canada	*CYP11A1* sequencing
13	74630968G>A	c. 1378C>T	R460W	rs535782968	2; 246170;8.124e-6	Missense*^a^*	ND	United Kingdom	*CYP11A1* sequencing

Abbreviations: NA, not applicable; ND, not determined, WES, whole exome sequencing.

^a^ Predicted consequence of variant at protein level.

The clinical details for the affected individuals are presented in Table 1. Diagnosis occurred at variable ages from 6 months to 16 years. In six children or young people (6 of 19; 32%), the diagnosis was facilitated by a history of PAI in their siblings. Hyperpigmentation was often present at the time of diagnosis (13 of 19; 68%), and many children had a history of ketotic hypoglycemia or hypoglycemic convulsions (10 of 19; 53%). All the children had been treated with glucocorticoid replacement, and almost one-half of them had received mineralocorticoid replacement (8 of 19; 42%). Most children had developed normally in puberty, although four boys experienced pubertal delay (four of nine postpubertal males; 44%), and mildly elevated gonadotropin concentrations were found in several adults.

The protein change resulting from the recurrent rs6161, p.Glu314Lys, was predicted to be benign by commonly used algorithms such as SIFT (Sorting Intolerant from Tolerant) and PolyPhen-2 but “disease causing” by MutationTaster2. Furthermore, the nucleotide change, in exon 5, is predicted to affect splicing (Human Splicing Finder 3.0; ESEfinder; Supplemental Table 7). The *trans* variants were all extremely rare, with MAF ranging from 0 to 2.2 × 10^−5^ in gnomAD (Table 2). In many cases, the variant resulted in an obviously deleterious effect; an early stop gain mutation (p.Arg120Ter, p.Arg439Ter, and p.Arg424Ter), a deletion or insertion causing frameshift and premature stop codon (c.566dupC, c.835delA, and c.790_802del), or a canonical splice site change likely to cause skipping of the adjacent exon and giving rise to a prematurely truncated mRNA (c.1158-2A>G and c.426-2A>G). These alleles are liable to be destroyed by nonsense-mediated mRNA decay (NMD). The missense variants p.Leu335Pro, seen in two siblings (family 5) and the p.Arg460Trp, seen in family 13, are in conserved residues and, on analysis, were predicted deleterious (SIFT) or probably damaging (PolyPhen). However, most intriguingly, two very rare synonymous variants were observed (c.990G>A and c.1173C>T), with no predicted amino acid change (Thr330 = and Ser391 =) in exons 5 and 7, respectively (Fig. 1). Both were designated “disease-causing” by MutationTaster2 and predicted to alter splicing (Human Splicing Finder, 3.0, results in Supplemental Tables 8 and 9).

### B. Investigation of Splicing

**Figure 2.**
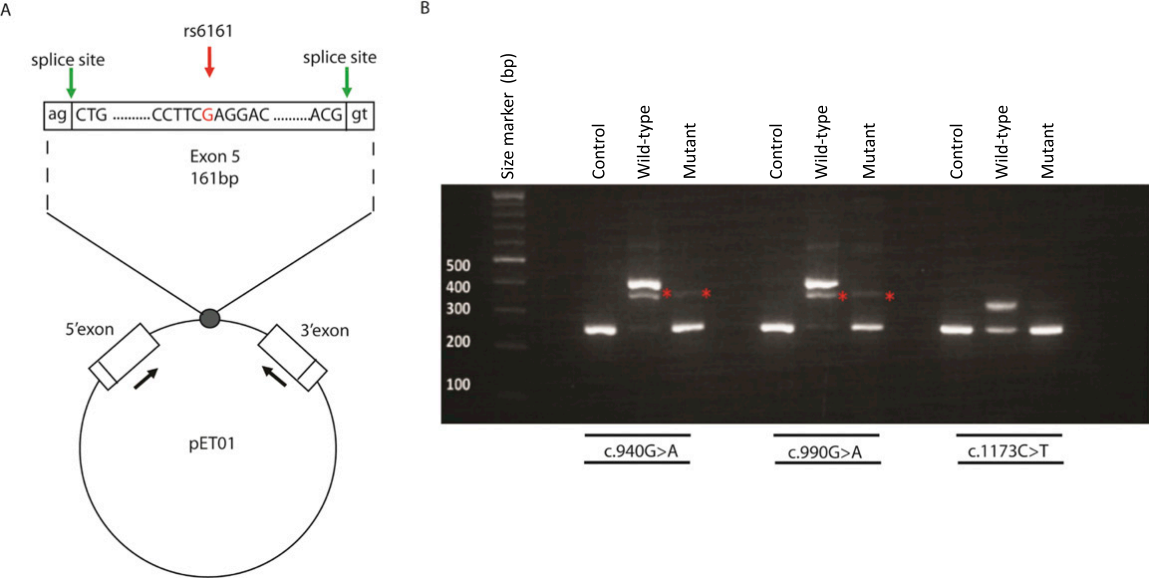
An *in vitro* assay revealed aberrant splicing of variants c.940G>A, c.990G>A, and c.1173C>T. (A) Minigene construction. Diagram of exon 5 and parts of flanking introns 4 and 5, inserted into the MCS of the pET01 construct (Lower). In the pET01 construct, the intron containing the MCS is flanked by the 5ʹ-donor and 3ʹ-acceptor splice sites of preproinsulin 5ʹ and 3ʹ exons, respectively (green arrows) (http://www.mobitec.com/cms/products/bio/04_vector_sys/exontrap.html). The expression of this vector sequence was driven by the promoter present in the long terminal repeat of Rous sarcoma virus, followed by a short stretch of a eukaryotic gene (phosphatase). The sequences containing the mutations detected in *CYP11A1* exons 5 and 7 (mutant) or those that did not (WT) were cloned into the MCS of pET01. The primers used in the RT-PCR experiments within the preproinsulin 5ʹ and 3ʹ exons are indicated by the black arrows (Supplemental Table 5). (B) Representative results of RT-PCR analysis using HEK293 cells transfected with an empty pET01 vector (empty vector), the pET01 vector containing the WT exon or the mutant exon: (Left) the c.940A in exon 5, (Middle) the c.990A change in exon 5, (Right) the C.1174T change in exon 7. A transcript of 225 bp was observed in the empty and mutant vector RT-PCRs, for all variants investigated, corresponding to the two-exon amplification product resulting from splicing of the preproinsulin 5′ and 3′ exons from the vector. For WT vectors containing c.940G and c.990G, a 386 bp transcript was observed corresponding to the three-exon amplification product resulting from correct splicing of *CYP11A1* exon 5 between the two vector exons. The intermediate band for exon 5 constructs, at ~350 bp (asterisk), was shown to be a mixture of a sequence that included both 386- and 225-bp bands. For WT c.1173C, a 304-bp transcript corresponding to the size of the three-exon amplification product resulting from correct splicing of *CYP11A1* exon 7 was observed. Sanger sequencing confirmed these findings (data not shown).

To investigate the potential splicing effects of these variants, a series of functional studies were undertaken. SNP rs6161 (c.940G>A) is within exon 5, 51 bp upstream of the natural splice donor site of intron 6; c.990G>A occurs at the last base of exon 5; and c.1173C>T is within exon 7, 16 bp downstream of natural acceptor site of intron 6 (Fig. 1). Using minigene constructs (Fig. 2A), the WT allele (c.940G) showed inclusion of exon 5, but the variant allele (c.940A) caused exon skipping (Fig. 2B). Similarly, we showed that variant c.990A caused exon 5 skipping, and variant c.1173T caused complete skipping of exon 7 (Fig. 2B). The WT exon 7, c.1173C, has also been reported to show a degree of exon skipping *in vitro*, consistent with the skipping of this exon in certain transcripts (CD013982) reported by Jin *et al.* [28]. In each instance, exon skipping would result in a frameshift and premature translation-termination codon, p.Ala277AspfsTer11 (for exon 5 variants) and p.Leu387HisfsTer29 (for exon 7 variant), if the message was not destroyed *in vivo* by NMD. Sanger sequencing of cDNA fragments confirmed the variant caused the skipping of its containing exon in each case, with no evidence of cryptic splice site usage (data not shown).

**Figure 3.**
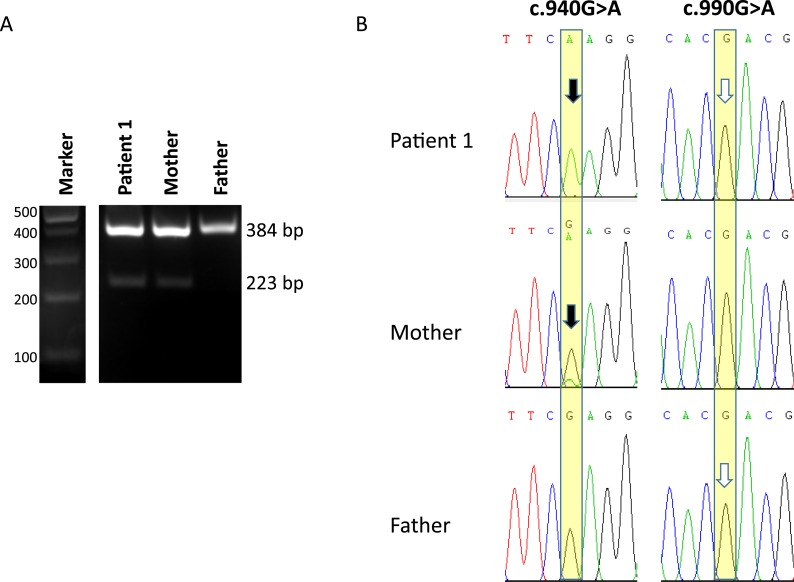
Sequence analysis of *in vivo* splicing in patient 1 and his parents. (A) RT-PCR amplification products using primers in exons 4 and 6 of *CYP11A1*. Lanes 1 and 2, patient 1 and his mother, showing two bands corresponding to transcripts containing exon 5 (upper band) and skipping exon 5 (lower band); this was confirmed by Sanger sequencing. In contrast, in lane 3, the father showed only the upper, exon 5 containing, transcript. (B) Partial chromatograms from Sanger sequencing of the upper bands in the patient, mother, and father. For the c.990G>A change (Right), the patient and father’s sequence revealed only the WT c.990G (white arrows), suggesting that the c.990A variant results in exon skipping and destruction of the truncated mRNA by NMD. For c.940G>A (Left), the patient’s sequence only has the mutant c.940A allele A inherited from his mother (black arrow), corroborating the NMD destruction of the c.990A allele inherited from his father; if it were present, it would give a heterozygous base at this position. In contrast, the mother’s sequence shows both WT G and a small peak of the mutant A (black arrow), consistent with skipping of mutant exon 5 in most mutant transcripts but revealing the presence of some transcripts containing exon 5.

To investigate the potential splice effects *in vivo*, we studied genomic DNA and RNA-derived cDNA in fibroblasts from subject 1 and his parents. By PCR of exon 5 in genomic DNA, it was confirmed that he had inherited the c.940G>A variant from his mother and the c.990G>A variant from his father (Fig. 3A). PCR of genomic DNA demonstrated that both alleles were equally represented (data not shown). However, at the RNA level, the absence of the c.990A allele was complete, with no exon skipped RNA detected by RT-PCR from exon 4 to 6 (Fig. 3A). Also, both the patient’s and the father’s sequences showed WT c.990G only (Fig. 3B). This finding would be consistent with NMD of the variant RNA species. In contrast, the c.940A allele showed evidence of skipping of exon 5, alongside production of a normally spliced, exon 5-containing, transcript. In the subject’s and his mother’s RNA, two bands were seen, the upper corresponding to exons 4-5-6 and the lower consisting of a transcript lacking exon 5 (bands at 384 and 223 bp, respectively, in Fig. 3A; confirmed by Sanger sequencing, data not shown). As expected, the patient’s RNA contained only the mutant c.940A transcript at this position, because the paternal “WT” allele had presumably been degraded by NMD. In the mother, both c.940A and c.940G transcripts were detected, with the c.940A less abundant than the WT, consistent with exon skipping and NMD for most of the variant allele (see the relative height of the peaks for the mutant “A” and WT “G” seen in the mother’s RNA; Fig. 3B, arrow).

### C. Assessment of Protein Function

**Figure 4.**
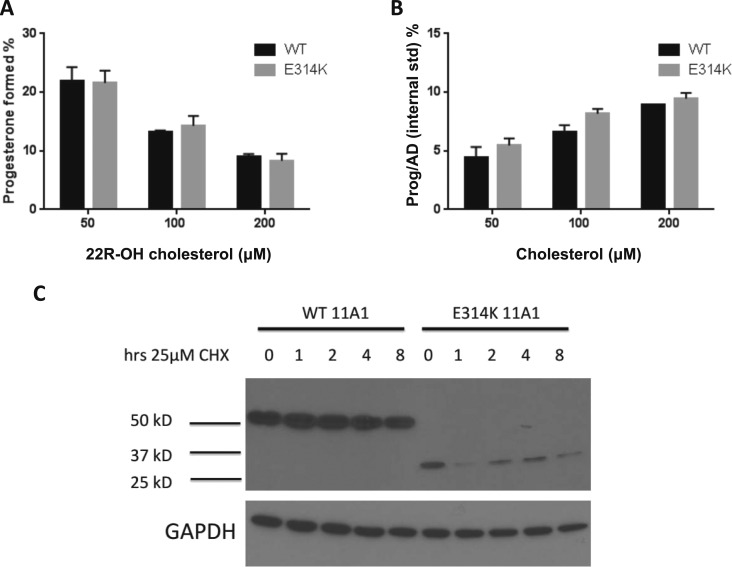
The p.314Lys protein evaluation. (A) Side-chain cleavage activity of CYP11A1 mutant p.314Lys was unaltered in *E. coli*. Expression of the purified mutant protein in *E. coli* showed comparable activity (black bars) as the WT enzyme (gray bars). The activity of the mutant p.314Lys was determined to be indistinguishable from WT protein, whether the substrate was 22R-hydroxycholesterol (Left) or cholesterol (Right). (B) The p.314Lys variant exhibited altered protein expression compared with WT when expressed in HEK-293T cells. HEK-293T cells were transiently transfected with either WT CYP11A1 or p.314Lys constructs and cultured for 48 hours. For the indicated times before protein collection, the cells were treated with 25 µM cycloheximide (CHX). Whole cell lysates were analyzed by immunoblot and probed with anti-CYP11A1 and anti–glyceraldehyde 3-phosphate dehydrogenase (GAPDH) antibodies. For the WT, a full-length protein was observed at 60 kDa. In contrast, for the p.314Lys mutant, the protein was truncated to 30 to 35 kDa, consistent with a cleavage event around the site of the amino acid change. CHX treatment revealed the mutant protein also had a shorter half-life.

Given the likelihood, therefore, that some of the c.940A (rs6161) transcripts will be translated into protein, we evaluated the function of the resultant mutant p.Glu314Lys protein. The mutation was re-created by site-directed mutagenesis, expressed in *E. coli*, and the catalytic activity of CYP11A1 was measured by assays of cholesterol and 22R-hydroxycholesterol conversion to pregnenolone. Despite the substitution of negatively charged Glu314 with a positively charged lysine residue, the mutant enzyme exhibited similar side-chain cleavage activities to WT CYP11A1 with either substrate (Fig. 4A and 4B). However, when cDNA encoding p.314Lys was transfected into HEK-293 cells, the protein was truncated (degraded), and the half-life after cycloheximide treatment was much shorter than that for WT (Fig. 4C). The truncated protein was consistent in size with potential proteolytic cleavage of the protein around the p.Glu314Lys change and, together with the splicing results, suggests relatively little functional CYP11A1 would be produced from this allele. To ascertain whether enough protein would escape degradation in intact cells to produce pregnenolone and to test for activity in the other two identified missense mutations, V79 cells were transfected with pcDNA3 expression plasmid encoding WT CYP11A1 or the three mutations. Pregnenolone formation from 22R-hydroxycholesterol was measured using tandem mass spectrometry. Mutation p.Arg460Trp demonstrated <1% of WT activity. In contrast, mutations p.Glu314Lys and p.Leu335Pro yielded no time-dependent pregnenolone formation (Table 3).

**Table 3.** CYP11A1 Activity in Transfected V79 Cells

	Pregnenolone Formed (pg/well)	
*CYP11A1*	6 h	24 h
Wild-type	23,238 ± 3919	48,113 ± 10369
R460W	105 ± 28	388 ± 226
L335P	84 ± 15	73 ± 19
E314K	83 ± 3	86 ± 30

## 3. Discussion

PAI is a life-threatening condition. Glucocorticoid replacement is central to the management of PAI, with some patients also requiring mineralocorticoid replacement. Approximately 20 different genetic causes of PAI have been identified to date (www.icped.org). However, in a substantial proportion of individuals, the cause is not currently known.

The enzyme CYP11A1 plays a key role in the initial steps of steroidogenesis by catalyzing the cleavage of cholesterol to pregnenolone. Children with marked disruption of this enzyme typically present with severe salt-losing adrenal insufficiency in the first few days of life and a complete block in gonadal steroidogenesis, with female-typical external genitalia in 46,XY infants or a lack of puberty in 46,XX girls [3–5, 9–14]. More recently, partial loss-of-function of CYP11A1 has been reported in children with delayed-onset adrenal insufficiency and hypospadias or glucocorticoid insufficiency alone [6–8]. The glucocorticoid pathway seems especially vulnerable to loss of CYP11A1 activity, potentially owing to the much greater molar concentrations of cortisol required, and even low levels of residual enzyme activity appear to be sufficient for fetal gonadal steroidogenesis (Supplemental Table 1) [6–8]. Similar findings have been shown for *STAR*, a related protein involved in facilitating cholesterol transfer into mitochondria, where complete disruption causes congenital lipoid adrenal hyperplasia affecting the adrenal and sex hormones, but partial dysfunction is associated with predominant glucocorticoid insufficiency [29, 30].

The present study included individuals or families with PAI for which the cause was unknown. In 13 families (19 affected individuals), massively parallel sequencing revealed the rs6161 (c.940G>A, p.Glu314Lys) variant in *CYP11A1* in compound heterozygosity with another very rare and/or deleterious variant. We showed that the rs6161 variant and two rare synonymous variants (Thr330 = and Ser391 =) can cause missplicing, resulting in absent or dysfunctional proteins and thereby contributing to the pathogenesis in PAI. In our cohort, compound heterozygosity for the c.940G>A with a second disruptive variant accounted for >20% of undiagnosed PAI and was one of the most common causes of familial PAI. Our observations are supported by the recent case report of compound heterozygosity for c.940G>A with a disruptive splice variant (c.425+1G>A) in a boy with an indolent presentation of PAI from 3 years of age and a history of hypospadias [14]. The relatively high MAF of rs6161 in the population (~ in 120 Europeans are carriers) suggests a natural “pool” of this variant is present in this population and can manifest a phenotype when combined with a very rare disruptive variant in *trans*. Four individuals in gnomAD (of 138,595 individuals or 277190 alleles) were homozygous for rs6161 (c.G>940A). However, it is not known whether this was associated with any adrenal phenotype.

Clinically, most affected individuals included in the present study were at the mild end of the spectrum, with a diagnosis of “familial glucocorticoid deficiency” or “Addison disease.” For most, onset occurred during childhood, rather than in the neonatal period, and hypoglycemic convulsions or ketotic hypoglycemia were a common feature. Others had a more indolent course during adolescence or had PAI diagnosed only because of a family history and mild symptoms, highlighting the importance of diagnosing PAI and investigating “at risk” individuals. Glucocorticoid insufficiency was common to all affected individuals; however, approximately one-half required mineralocorticoid replacement, and disordered sex development did not occur. Four males experienced delayed puberty; however, the others had progressed through puberty normally, and two older men had fathered children. However, monitoring of mineralocorticoid activity and gonadal function will be needed for long-term management, and more detailed long-term studies are required to understand the natural history of this condition. Heterozygous parents appear unaffected, as usual for disorders of steroidogenesis.

The rs6161 allele alters splicing of the pre-mRNA sequence, with the result that many RNA transcripts do not include exon 5. The transcripts that do include exon 5 will result in a mutant protein with an amino acid change, glutamic acid to lysine, at residue 314. Although the mutation reverses the charge on this side chain from negative to positive, the residue is located on the surface of the protein in the I-helix facing solvent and has no effect on protein function. Given this ambiguity, we subsequently showed that the purified p.314Lys mutation has normal spectral and catalytic properties in *E. coli* but results in an unstable truncated protein of 30 to 35 kDa when expressed in mammalian cells. *In vivo*, in affected individuals, it is likely that the combination of defective splicing and protein dysfunction will result in low levels of functional, full-length protein derived from this allele. This might be sufficient for gonadal steroidogenesis but insufficient for glucocorticoid production. Furthermore, our studies have only been possible in skin fibroblasts or nonsteroidogenic cells; thus, it is possible that greater residual function occurs in the adrenal gland or gonad.

Tools for the prediction of the functional effects of nonsynonymous variants are well-established [31]; however, these might not predict the effect such changes will have on splicing. In particular, variants causing benign amino acid changes might have their effect on mRNA processing or post-translational modification. Synonymous or “silent” changes are often ignored and, when tested, the results from commonly used prediction tools might prove inconclusive. Up to 45% of synonymous SNPs are likely to alter pre-mRNA splicing, and regulatory information could be dispersed throughout nearly every nucleotide in an exon [32, 33], making it important to consider them in variant analyses, especially when very rare and discovered in known or likely causative genes.

## 4. Conclusion

We have concluded that the rs6161 change, in conjunction with another loss-of-function allele, is responsible for a substantial proportion of unsolved PAI, especially in European populations. The results of the present study have highlighted the limitations of relying on common *in silico* prediction tools and the necessity for individual assessment of a polymorphism. This assessment should consider, not only the protein function in a suitable cell line and assay system, but also the consequences of the change at the nucleic acid level.

## Supplementary Material

Supplemental Table

## Appendix

### Web Resources

Human Splicing Finder 3.0: http://www.umd.be/HSF3/

Mutation Taster: http://www.mutationtaster.org/

PolyPhen-2: http://genetics.bwh.harvard.edu/pph2/

Sorting Intolerant from Tolerant (SIFT): http://provean.jcvi.org/genome_submit_2.php

dbSNP: www.ncbi.nlm.nih.gov/SNP/

Exome Aggregation Consortium (ExAC), Cambridge, MA: http://exac.broadinstitute.org; accessed February 2018

The Genome Aggregation Database: http://gnomad.broadinstitute.org/; accessed February 2018

Variant effect predictor: http://www.ensembl.org/Tools/VEP

Online Mendelian Inheritance in Man (OMIM): http://www.omim.org/

The Human Gene Mutation Database: http://www.hgmd.cf.ac.uk/ac/index.php

Ingenuity variant analysis: http://www.ingenuity.com/

Otogenetics: http://www.otogenetics.com/

ESEfinder 3.0: http://krainer01.cshl.edu/cgi-bin/tools/ESE3/esefinder.cgi?process=home

## Abbreviations:

Adx: adrenodoxin
CYP11A1: cytochrome P450 side chain cleavage enzyme
dNTP: deoxyribonucleotide triphosphate
FBS: fetal bovine serum
gnomAD: Genome Aggregation Database
MAF: minor allele frequency
MCS: multiple cloning site
NMD: nonsense-mediated mRNA decay
PAI: primary adrenal insufficiency
SNP: single nucleotide polymorphism
SNV: single nucleotide variant
WT: wild type

## References

[1] Miller WL, Auchus RJ The molecular biology, biochemistry, and physiology of human steroidogenesis and its disorders. Endocr Rev. 2011;32(1):81–151. 21051590 10.1210/er.2010-0013 PMC3365799

[2] Miller WL Why nobody has P450scc (20,22 desmoslase) deficiency. J Clin Endocrinol Metab. 1998;83(4):1399–1400. 9543177 10.1210/jcem.83.4.4734-7

[3] Guran T, Buonocore F, Saka N, Ozbek MN, Aycan Z, Bereket A, Bas F, Darcan S, Bideci A, Guven A, Demir K, Akinci A, Buyukinan M, Aydin BK, Turan S, Agladioglu SY, Atay Z, Abali ZY, Tarim O, Catli G, Yuksel B, Akcay T, Yildiz M, Ozen S, Doger E, Demirbilek H, Ucar A, Isik E, Ozhan B, Bolu S, Ozgen IT, Suntharalingham JP, Achermann JC Rare causes of primary adrenal insufficiency: genetic and clinical characterization of a large nationwide cohort. J Clin Endocrinol Metab. 2016;101(1):284–292. 26523528 10.1210/jc.2015-3250 PMC4701852

[4] Tee MK, Abramsohn M, Loewenthal N, Harris M, Siwach S, Kaplinsky A, Markus B, Birk O, Sheffield VC, Parvari R, Hershkovitz E, Miller WL Varied clinical presentations of seven patients with mutations in CYP11A1 encoding the cholesterol side-chain cleavage enzyme, P450scc. J Clin Endocrinol Metab. 2013;98(2):713–720. 23337730 10.1210/jc.2012-2828 PMC3565115

[5] Parajes S, Chan AO, But WM, Rose IT, Taylor AE, Dhir V, Arlt W, Krone N Delayed diagnosis of adrenal insufficiency in a patient with severe penoscrotal hypospadias due to two novel P450 side-change cleavage enzyme (CYP11A1) mutations (p.R360W; p.R405X). Eur J Endocrinol. 2012;167(6):881–885. 22968487 10.1530/EJE-12-0450 PMC3494866

[6] Parajes S, Kamrath C, Rose IT, Taylor AE, Mooij CF, Dhir V, Grötzinger J, Arlt W, Krone N A novel entity of clinically isolated adrenal insufficiency caused by a partially inactivating mutation of the gene encoding for P450 side chain cleavage enzyme (CYP11A1). J Clin Endocrinol Metab. 2011;96(11):E1798–E1806. 21880796 10.1210/jc.2011-1277

[7] Sahakitrungruang T, Tee MK, Blackett PR, Miller WL Partial defect in the cholesterol side-chain cleavage enzyme P450scc (CYP11A1) resembling nonclassic congenital lipoid adrenal hyperplasia. J Clin Endocrinol Metab. 2011;96(3):792–798. 21159840 10.1210/jc.2010-1828 PMC3047228

[8] Rubtsov P, Karmanov M, Sverdlova P, Spirin P, Tiulpakov A A novel homozygous mutation in CYP11A1 gene is associated with late-onset adrenal insufficiency and hypospadias in a 46,XY patient. J Clin Endocrinol Metab. 2009;94(3):936–939. 19116240 10.1210/jc.2008-1118

[9] Kim CJ, Lin L, Huang N, Quigley CA AvRuskin TW, Achermann JC, Miller WL Severe combined adrenal and gonadal deficiency caused by novel mutations in the cholesterol side chain cleavage enzyme, CYP11A1. J Clin Endocrinol Metab. 2008;93:696–702. 18182448 10.1210/jc.2007-2330 PMC2266942

[10] al Kandari H, Katsumata N, Alexander S, Rasoul MA Homozygous mutation of P450 side-chain cleavage enzyme gene (CYP11A1) in 46, XY patient with adrenal insufficiency, complete sex reversal, and agenesis of corpus callosum. J Clin Endocrinol Metab. 2006;91(8):2821–2826. 16705068 10.1210/jc.2005-2230

[11] Hiort O, Holterhus PM, Werner R, Marschke C, Hoppe U, Partsch CJ, Riepe FG, Achermann JC, Struve D Homozygous disruption of P450 side-chain cleavage (CYP11A1) is associated with prematurity, complete 46,XY sex reversal, and severe adrenal failure. J Clin Endocrinol Metab. 2005;90(1):538–541. 15507506 10.1210/jc.2004-1059

[12] Katsumata N, Ohtake M, Hojo T, Ogawa E, Hara T, Sato N, Tanaka T Compound heterozygous mutations in the cholesterol side-chain cleavage enzyme gene (CYP11A) cause congenital adrenal insufficiency in humans. J Clin Endocrinol Metab. 2002;87(8):3808–3813. 12161514 10.1210/jcem.87.8.8763

[13] Tajima T, Fujieda K, Kouda N, Nakae J, Miller WL Heterozygous mutation in the cholesterol side chain cleavage enzyme (p450scc) gene in a patient with 46,XY sex reversal and adrenal insufficiency. J Clin Endocrinol Metab. 2001;86(8):3820–3825. 11502818 10.1210/jcem.86.8.7748

[14] Lara-Velazquez M, Perdomo-Pantoja A, Blackburn PR, Gass JM, Caulfield TR, Atwal PS A novel splice site variant in CYP11A1 in trans with the p.E314K variant in a male patient with congenital adrenal insufficiency. Mol Genet Genomic Med. 2017;5(6):781–787. 29178636 10.1002/mgg3.322 PMC5702577

[15] Chan LF, Campbell DC, Novoselova TV, Clark AJ, Metherell LA Whole-exome sequencing in the differential diagnosis of primary adrenal insufficiency in children. Front Endocrinol (Lausanne). 2015;6:113. 26300845 10.3389/fendo.2015.00113 PMC4525066

[16] Benoit I, Drui D, Chaillous L, Dupas B, Mosnier JF, Charbonnel B, Cariou B A corticotroph pituitary adenoma as the initial presentation of familial glucocorticoid deficiency. Eur J Endocrinol. 2009;161(1):195–199. 19423561 10.1530/EJE-09-0100

[17] Poliandri A, Miller D, Howard S, Nobles M, Ruiz-Babot G, Harmer S, Tinker A, McKay T, Guasti L, Dunkel L Generation of kisspeptin-responsive GnRH neurons from human pluripotent stem cells. Mol Cell Endocrinol. 2017;447:12–22. 28232089 10.1016/j.mce.2017.02.030

[18] Harikrishna JA, Black SM, Szklarz GD, Miller WL Construction and function of fusion enzymes of the human cytochrome P450scc system. DNA Cell Biol. 1993;12(5):371–379. 8517924 10.1089/dna.1993.12.371

[19] Woods ST, Sadleir J, Downs T, Triantopoulos T, Headlam MJ, Tuckey RC Expression of catalytically active human cytochrome p450scc in *Escherichia coli* and mutagenesis of isoleucine-462. Arch Biochem Biophys. 1998;353(1):109–115. 9578606 10.1006/abbi.1998.0621

[20] Tuckey RC, Li W, Shehabi HZ, Janjetovic Z, Nguyen MN, Kim TK, Chen J, Howell DE, Benson HA, Sweatman T, Baldisseri DM, Slominski A Production of 22-hydroxy metabolites of vitamin d3 by cytochrome p450scc (CYP11A1) and analysis of their biological activities on skin cells. Drug Metab Dispos. 2011;39(9):1577–1588. 21677063 10.1124/dmd.111.040071 PMC3164270

[21] Yoshimoto FK, Zhou Y, Peng HM, Stidd D, Yoshimoto JA, Sharma KK, Matthew S, Auchus RJ Minor activities and transition state properties of the human steroid hydroxylases cytochromes P450c17 and P450c21, from reactions observed with deuterium-labeled substrates. Biochemistry. 2012;51(36):7064–7077. 22873692 10.1021/bi300895w PMC3471366

[22] Black SM, Szklarz GD, Harikrishna JA, Lin D, Wolf CR, Miller WL Regulation of proteins in the cholesterol side-chain cleavage system in JEG-3 and Y-1 cells. Endocrinology. 1993;132(2):539–545. 8425475 10.1210/endo.132.2.8425475

[23] RRID:AB_2747382.

[24] RRID:AB_2107426.

[25] RRID:AB_330924.

[26] RRID:AB_2099233.

[27] Turcu AF, Nanba AT, Chomic R, Upadhyay SK, Giordano TJ, Shields JJ, Merke DP, Rainey WE, Auchus RJ Adrenal-derived 11-oxygenated 19-carbon steroids are the dominant androgens in classic 21-hydroxylase deficiency. Eur J Endocrinol. 2016;174(5):601–609. 26865584 10.1530/EJE-15-1181 PMC4874183

[28] Jin P, Fu GK, Wilson AD, Yang J, Chien D, Hawkins PR, Au-Young J, Stuve LL PCR isolation and cloning of novel splice variant mRNAs from known drug target genes. Genomics. 2004;83(4):566–571. 15028279 10.1016/j.ygeno.2003.09.023

[29] Baker BY, Lin L, Kim CJ, Raza J, Smith CP, Miller WL, Achermann JC Nonclassic congenital lipoid adrenal hyperplasia: a new disorder of the steroidogenic acute regulatory protein with very late presentation and normal male genitalia. J Clin Endocrinol Metab. 2006;91(12):4781–4785. 16968793 10.1210/jc.2006-1565 PMC1865081

[30] Metherell LA, Naville D, Halaby G, Begeot M, Huebner A, Nürnberg G, Nürnberg P, Green J, Tomlinson JW, Krone NP, Lin L, Racine M, Berney DM, Achermann JC, Arlt W, Clark AJ Nonclassic lipoid congenital adrenal hyperplasia masquerading as familial glucocorticoid deficiency. J Clin Endocrinol Metab. 2009;94(10):3865–3871. 19773404 10.1210/jc.2009-0467 PMC2860769

[31] Tang H, Thomas PD Tools for predicting the functional impact of nonsynonymous genetic variation. Genetics. 2016;203(2):635–647. 27270698 10.1534/genetics.116.190033 PMC4896183

[32] Mueller WF, Larsen LS, Garibaldi A, Hatfield GW, Hertel KJ The silent sway of splicing by synonymous substitutions. J Biol Chem. 2015;290(46):27700–27711. 26424794 10.1074/jbc.M115.684035 PMC4646019

[33] Julien P, Miñana B, Baeza-Centurion P, Valcárcel J, Lehner B The complete local genotype-phenotype landscape for the alternative splicing of a human exon. Nat Commun. 2016;7:11558. 27161764 10.1038/ncomms11558 PMC4866304

