# Supplementary material for: Predicted Benign and Synonymous Variants in CYP11A1 Cause Primary Adrenal Insufficiency Through Missplicing
Source: J Endocr Soc. 2018 Oct 30;3(1):201–21. doi: 10.1210/js.2018-00130 (PMC6316989; doi:10.1210/js.2018-00130)

**Supplementary data.**

**Supplementary Table 1. Previously described children with *CYP11A1* mutations**

| **Mutation at protein level** | **Number of cases** | **Age(s) at presentation** | **Salt-wasting** | **Aldosterone levels** | **DSD** | **Reference** | **Comments** |
| --- | --- | --- | --- | --- | --- | --- | --- |
| [Arg451Trp];[Arg451Trp] | 9 | 1-6y | 6 out of 9 | low in half | “micropenis” in 1 | [3] |  |
| [Arg232*];[Arg232*] | 2 | neonates | yes | nil | no | [4] |  |
| [Arg232*];[Phe215Ser] | 4 | 1.2-4.75y | yes | nil | 1 with small penis | [4] |  |
| [Arg120*];[Arg120*] | 1 | neonate | yes | nil | no | [4] |  |
| [Arg360Trp];[Arg405*] | 1 | neonate | no | low | yes | [5] |  |
| [Arg451Trp];[Arg451Trp] | 2 | 2.5-2.8 | yes | low | no | [6] |  |
| [Iso279Tyrfs*10];[Ala269Val] | 2 | 4 and 9y | no data | no data | yes in male patient | [7] | described as 'like non-classic LCAH' |
| [Leu222Pro];[Leu222Pro] | 1 | 9y | yes? | low | hypospadias | [8] |  |
| [Leu141Trp];[Val415Glu] | 1 | neonate | yes | low | yes | [9] |  |
| [Iso279Tyrfs*10]; [K142Nfs*2] | 1 | neonate | yes | low | yes | [9] | K142Nfs*2 previously described as [IVS3+(2-3)insT |
| [Ala359Val];[Ala359Val] | 1 | 1.75y | yes | low | yes | [10] |  |
| [Iso279Tyrfs*10];[Iso279Tyrfs*10] | 1 | neonate | yes | low | yes | [11] |  |
| [Ala189Val];[Arg353Trp] | 1 | 7m | no | normal | no | [12] | Ala189Val is a splicing mutation |
| [Asp271_Val272insGlyAsp];[?] | 1 | 4y | no data | normal | yes | [13] | haploinsufficiency hypothesized |
| [Glu91_Lys142del];[Glu314Lys] | 1 | 12y | no data | No data but treated with fludrocortisone | hypospadias | [14] |  |

All patients had cortisol deficiency. DSD, disorder/difference in sex development.

**Supplementary Table 2. Summary of the prevalence of primary adrenal insufficiency associated with *CYP11A1* c940G>A in the cohorts studied and amongst individuals where the cause is currently unknown.**

| **Cohort** | **Genetic cause** | **^a^All individual patients** | **^b^Families without a diagnosis** |
| --- | --- | --- | --- |
|  |  | n (%) | n (%) |
| **QMUL/Barts/RLH** | ^c^Cause identified | 213 (83.2) |  |
|  | ^d^*CYP11A1* c.940G>A | 8 (3.1) | 7 |
|  | ^e^Unknown | 35 (13.7) | 23 |
|  | **Total** | **256 (100)** | **30 (100)** |
|  | % unknown due to *CYP11A1* c.940G>A | 8/43 (18.6) | 7/30 (23.3) |
| **UCL/GOSH** | Cause identified | 32 (56.1) |  |
|  | *CYP11A1* c.940G>A | 7 (2.3) | 4 |
|  | Unknown | 18 (31.6) | 16 |
|  | **Total** | **57 (100)** | **20 (100)** |
|  | % unknown due to *CYP11A1* c.940G>A | 7/25 (28.0) | 4/20 (20.0) |
| **Turkish** | Cause identified | 73 (89.0) |  |
|  | CYP11A1 c.940G>A | 1 (1.2) | 1 (11.1) |
|  | Unknown | 8 (9.8) | 8 (88.9) |
|  | **Total** | **82 (100)** | **9 (100)** |
|  | % unknown due to CYP11A1 c.940G>A | 1/9 (11.1) | 1/9 (11.1) |
| **Total** | % due to CYP11A1 c.940G>A | **16/395 (4.1)** |  |
| **Unknown** | % due to CYP11A1 c.940G>A | **16/77 (20.8)** | **12/59 (20.3)** |
|  | gnoMAD MAF | 0.0026 | 0.0026 |
|  | Our cohorts MAF | 0.0203 | 0.0102 |

Three cohorts were considered in this study; QMUL/Barts/RLH = Queen Mary University/St Bartholomew’s and Royal London Hospitals; UCL/GOSH = University College London/ Great Ormond Street Hospital and Turkish = patients from various centres in Turkey; MAF, minor allele frequency; ^a^Data represent all patients with PAI. In families with two or three affected individuals, all of them are included in the total numbers shown. ^b^Data represent individual families where there is at least one member with PAI; ^c^indicates patients in whom a genetic diagnosis had been identified prior to this study; ^d^indicates patients in whom the c.940G>A variant was discovered in this study; ^e^indicates patients in whom a genetic diagnosis has not been identified.

**Supplementary Table 3. Primers for amplification of *CYP11A1* exons from genomic DNA**

| **PRIMER NAME** | **Forward sequence** | **Reverse sequence** |
| --- | --- | --- |
| *CYP11A1 exon 1* | CAGAAATTCCAGACTGAACC | GAAGTTAGACAGGAGTTTGG |
| *CYP11A1 exon 2* | TGTATCCATAGCTCTCCTCG | CTCTGCCCTCTCCACAG |
| *CYP11A1 exon 3* | AAAACTGAGTCAGCCCC | GAGTGAACACTGAGTCCTCC |
| *CYP11A1 exon 4* | GAGTTGTGGCTCTCAGATG | ACATAGCGTGGGACAAAG |
| *CYP11A1 exon 5* | GTGCCACCTTTCACCAC | AACAGGGTTTCAGACAACG |
| *CYP11A1 exons 6 and 7* | AATTTACCATGGGGTCAAG | CACCCTCTGTCTGCAATTC |
| *CYP11A1 exon 8* | GGGTTTTGTGCTCAGGG | AAGATTGGTGCCTTCATTAG |

**Supplementary Table 4. Primers for amplification of *CYP11A1* exons 5 and 7 and surrounding intronic sequence from genomic DNA**

| **PRIMER** | **Forward sequence** | **Reverse sequence** |
| --- | --- | --- |
| E314K | AGCTATA**TCTAGA**TTACAGCCATGTGACCTT | AGCTATA**TCTAGA**ATGAGTAGGAACGCCTT |
| T330= | AGCTATA**TCTAGA**TTGGTGCCACCTTTCAC | AGCTATA**TCTAGA**TCCTCTCTCACAGGCAA |
| S391= | AGCTATA**TCTAGA**TTACGAGGATGGGCTGG | AGCTATA**TCTAGA**ATGAAGTCTCCCTCCTAA |

Restriction enzyme sites for *Xba*I highlighted in bold font.

**Supplementary Table 5. Primers for generation and amplification of cDNA from ExonTrap vector.**

| **PRIMER** | **Sequence** |
| --- | --- |
| *cDNA-primer 01* | GATCCACGATGC |
| *PCR primer 02* | GAGGGATCCGCTTCCTGGCCC |
| *PCR primer 03* | CTCCCGGGCCACCTCCAGTGCC |

**Supplementary Table 6. Primers for amplification of cDNA from human samples.**

| **Primer** | **Sequence** |
| --- | --- |
| CYP11A1 Exon 3 | ATGCCATCTACCAGATGTTC |
| CYP11A1 Exon 6 | ACCAGCTGTAGCATCGTG |

**Predictions of the effect(s) of CYP11A1 variants on splicing from HSF3.0**

**Supplementary Table 7.** rs6161 – breaks an SC35=SRSF2 site (exon splice enhancer [ESE]) creates a new SRp40 site (exon splice silencer [ESS])


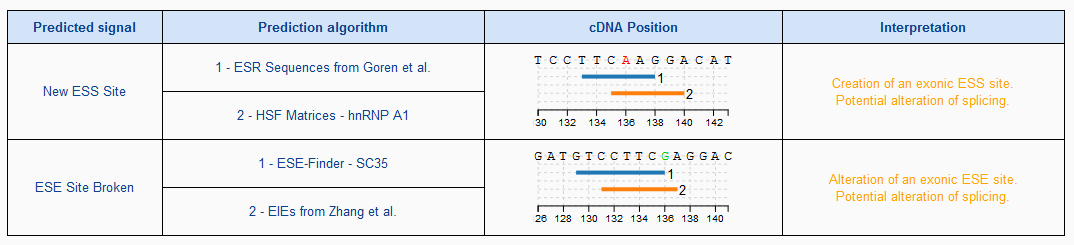


**Supplementary Table 8.** c.990G>A (Thr330=) – breaks/moves SF2/ASF site and creates a new acceptor site next door to natural donor


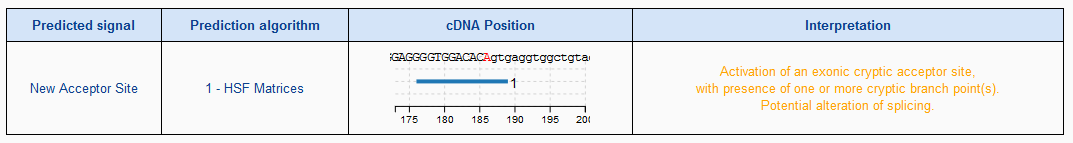


**Supplementary Table 9.** c.1173C>T (Ser391=) – creates a new ESS site, activates a cryptic acceptor site 18bp downstream of original acceptor.


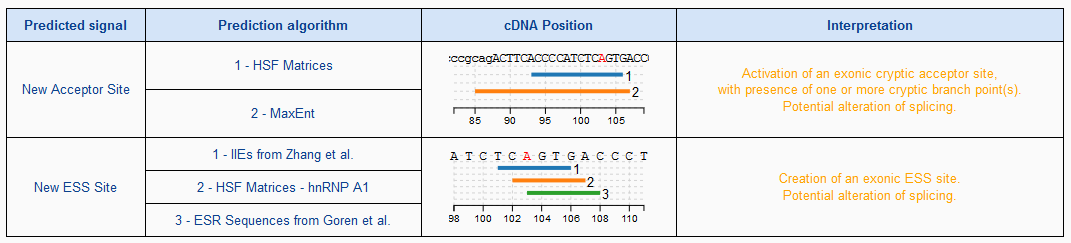

Supplement: Supplemental Table [file js.2018-00130.st1.docx]
